# Unraveling multi‐scale neuroimaging biomarkers and molecular foundations for schizophrenia: A combined multivariate pattern analysis and transcriptome‐neuroimaging association study

**DOI:** 10.1111/cns.14906

**Published:** 2024-08-08

**Authors:** Yanmin Peng, Chao Chai, Kaizhong Xue, Jie Tang, Sijia Wang, Qian Su, Chongjian Liao, Guoshu Zhao, Shaoying Wang, Nannan Zhang, Zhihui Zhang, Minghuan Lei, Feng Liu, Meng Liang

**Affiliations:** ^1^ School of Medical Imaging and Tianjin Key Laboratory of Functional Imaging Tianjin Medical University Tianjin China; ^2^ Department of Radiology and Tianjin Key Laboratory of Functional Imaging Tianjin Medical University General Hospital Tianjin China; ^3^ Department of Radiology, School of Medicine, Tianjin First Central Hospital Nankai University Tianjin China; ^4^ Department of Radiology and Nuclear Medicine, Xuanwu Hospital Capital Medical University Beijing China; ^5^ Department of Molecular Imaging and Nuclear Medicine Tianjin Medical University Cancer Institute and Hospital Tianjin China

**Keywords:** gene expression, multiscale analysis, multivariate pattern analysis, regional homogeneity, schizophrenia

## Abstract

**Aims:**

Schizophrenia is characterized by alterations in resting‐state spontaneous brain activity; however, it remains uncertain whether variations at diverse spatial scales are capable of effectively distinguishing patients from healthy controls. Additionally, the genetic underpinnings of these alterations remain poorly elucidated. We aimed to address these questions in this study to gain better understanding of brain alterations and their underlying genetic factors in schizophrenia.

**Methods:**

A cohort of 103 individuals with diagnosed schizophrenia and 110 healthy controls underwent resting‐state functional MRI scans. Spontaneous brain activity was assessed using the regional homogeneity (ReHo) metric at four spatial scales: voxel‐level (Scale 1) and regional‐level (Scales 2–4: 272, 53, 17 regions, respectively). For each spatial scale, multivariate pattern analysis was performed to classify schizophrenia patients from healthy controls, and a transcriptome‐neuroimaging association analysis was performed to establish connections between gene expression data and ReHo alterations in schizophrenia.

**Results:**

The ReHo metrics at all spatial scales effectively discriminated schizophrenia from healthy controls. Scale 2 showed the highest classification accuracy at 84.6%, followed by Scale 1 (83.1%) and Scale 3 (78.5%), while Scale 4 exhibited the lowest accuracy (74.2%). Furthermore, the transcriptome‐neuroimaging association analysis showed that there were not only shared but also unique enriched biological processes across the four spatial scales. These related biological processes were mainly linked to immune responses, inflammation, synaptic signaling, ion channels, cellular development, myelination, and transporter activity.

**Conclusions:**

This study highlights the potential of multi‐scale ReHo as a valuable neuroimaging biomarker in the diagnosis of schizophrenia. By elucidating the complex molecular basis underlying the ReHo alterations of this disorder, this study not only enhances our understanding of its pathophysiology, but also pave the way for future advancements in genetic diagnosis and treatment of schizophrenia.

## Graphical abstract

Our study highlights the potential of multi‐scale ReHo analysis as a valuable neuroimaging biomarker in the diagnosis of schizophrenia. Furthermore, we observed spatial scale‐dependent differences in enriched biological processes in the brain associated with schizophrenia. These related genes were mainly linked to immune responses, inflammation, synaptic signaling, ion channels, cellular development, myelination, and transporter activity.

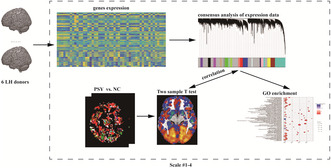

## 1 INTRODUCTION

Schizophrenia is a chronic, heterogeneous, devastating mental disorder characterized by symptoms such as delusions, hallucinations, reduced emotional expression, diminished motivation, and cognitive impairments.^1^, ^2^ It affects approximately 1% of the global population and places a significant burden on healthcare systems worldwide.^3^ Moreover, individuals with schizophrenia experience a decreased life expectancy, with reports indicating that the average lifespan of some individuals with schizophrenia is approximately 15 years shorter than that of the general population.^4^ Currently, the diagnosis of schizophrenia relies solely on clinical evaluation which can be influenced by subjective judgment and thus results in lower diagnostic reliability.^5^ Hence, the discovery of distinct biomarkers for precise schizophrenia diagnosis is crucial.

Over the last decade, resting‐state functional magnetic resonance imaging (fMRI) has emerged as a promising avenue to investigate the neurobiological underpinnings of schizophrenia. Regional homogeneity (ReHo), a metric reflecting local synchronization of spontaneous neural activity,^6^ is widely recognized as a robust biomarker for psychosis,^7^ providing invaluable insights into regional temporal synchronization within the brain.^8^ Reduced ReHo in resting‐state studies indicates a disruption in local synchrony within spontaneous neuronal activity, emphasizing its role as a fundamental neuropathological hallmark of schizophrenia.^9^, ^10^, ^11^, ^12^ Current studies on ReHo in schizophrenia primarily concentrate on voxel‐level spatial scales, representing a fine‐grained, local view.^9^, ^10^, ^11^, ^12^ This approach, while informative, introduces higher levels of signal noise, potentially adversely impacting classification accuracy.^13^ Concurrently, a few studies have also attempted to investigate the classification accuracy of schizophrenia at the region level using ReHo with different brain parcellation schemes.^14^, ^15^ As spatial scales expand, larger voxels can enhance the signal noise ratio (SNR) while potentially losing some information. Various spatial scales contain varying amounts of information, influenced by the trade‐off between information loss and SNR improvement. Consequently, an important and often overlooked question remains: at what spatial scales do neural activity patterns in individuals with schizophrenia diverge from those in healthy subjects? Does discriminability vary across these different spatial scales, and which specific scale offers the highest efficacy for classifying schizophrenia?

Schizophrenia exhibits an estimated heritability of approximately 80%, highlighting the substantial genetic influence on the disorder.^16^, ^17^, ^18^ Extensive research has uncovered numerous genetic risk factors associated with schizophrenia, although the exact mechanisms behind the disease remain intricate and multifaceted, emphasizing the significant role of genetic factors in schizophrenia.^19^, ^20^ Nonetheless, the genetic mechanisms behind ReHo differences in schizophrenia remain largely unclear, and conventional genome‐wide association studies cannot identify the associated genetic variants. With the advancement of the Allen Human Brain Atlas (AHBA, http://human.brain‐map.org),^21^, ^22^ transcriptome‐neuroimaging association analysis has the potential to reveal the molecular basis of neuroimaging changes.^23^, ^24^, ^25^ However, to date, no transcriptome‐neuroimaging association study has been conducted to identify genes associated with ReHo alterations in schizophrenia, let alone across different spatial scales. Conducting such research is crucial, as it significantly enhances our comprehension of the molecular foundations of schizophrenia.

Building on prior research, the primary objectives of our present study can be summarized in two aspects. First, our aim was to distinguish individuals with schizophrenia from healthy subjects by utilizing the ReHo metric across various spatial scales, including voxel‐level and three region‐level scales. Second, a transcriptome‐neuroimaging association analysis was conducted to link transcriptome data from the AHBA database with the observed case–control ReHo changes in schizophrenia.

## 2 MATERIALS AND METHODS

### 2.1 Participants

The study received approval from the Ethics Committee of Tianjin Medical University General Hospital, and all subjects provided written informed consent before participating. A total of 103 patients with schizophrenia were recruited from Tianjin Medical University General Hospital. The diagnosis of schizophrenia was established through the consensus of two psychiatrists employing the Structured Clinical Interview for the DSM‐IV (SCID, patient edition). To assess the severity of clinical symptoms, the Positive and Negative Symptom Scale (PANSS) was utilized. Exclusion criteria involved the following: the presence of MRI contraindications, an inability to undergo MRI examinations, systemic medical conditions (such as cardiovascular disease, diabetes mellitus, cognitive impairment, cerebral stroke, hemorrhage, epilepsy, and tumors), congenital cerebral structural abnormalities, a history of head trauma, central nervous system (CNS) disorders, or substance abuse. In addition, a group of 110 age‐ and gender‐matched healthy controls (HCs) was recruited from nearby communities and assessed using the SCID non‐patient version to ensure an absence of any psychiatric disorder history. None of these healthy control participants had a history of psychotic episodes in their first‐degree relatives.

### 2.2 Methodology overview

**FIGURE 1:**
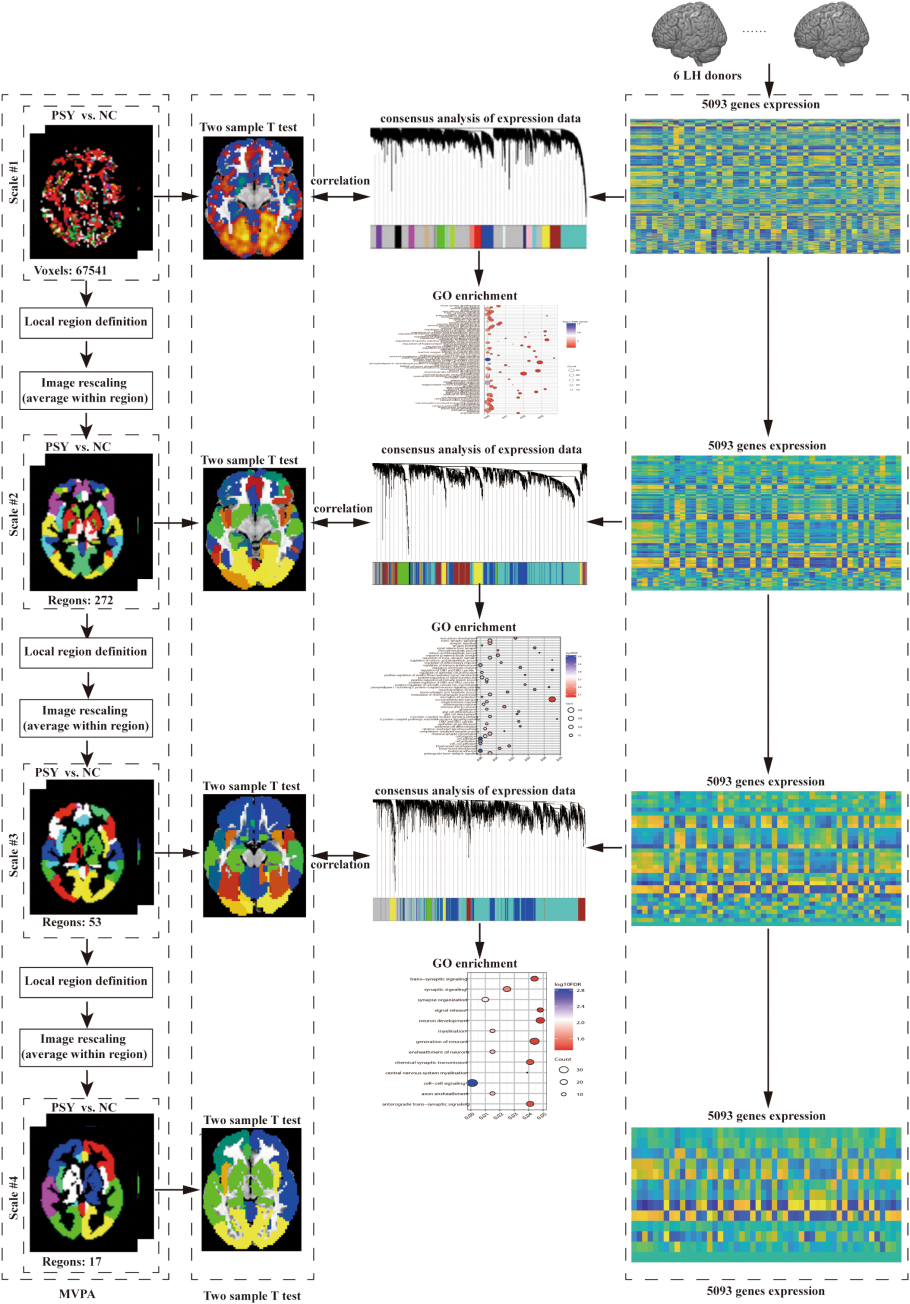
The flowchart of the spatial‐scale analysis to acquire the information of classifying schizophrenia from HCs and to characterize the involved gene expression profiles correlated with ReHo difference in schizophrenia at each spatial scale.

The framework, as shown in Figure 1, consists of two main components: (1) classification analysis and (2) transcriptome‐neuroimaging association analysis. In the first part, a multivariate pattern analysis (MVPA)^26^ was conducted to distinguish individuals with schizophrenia from HCs using the ReHo metric at various spatial scales, including voxel‐level, 272 regions, 53 regions, and 17 regions. In the second part, ReHo difference maps between individuals with schizophrenia and HCs at each spatial scale were generated through two‐sample *t*‐tests. Subsequently, gene expression data from the AHBA database were obtained, and a weighted gene co‐expression network analysis (WGCNA) was performed to investigate the relationships between ReHo difference maps and gene expression data. Finally, Toppogene (https://toppgene.cchmc.org/) was utilized to conduct enrichment analysis for genes correlated with ReHo difference maps in schizophrenia at each spatial scale.

### 2.3 Imaging data acquisition and preprocessing

A 3.0‐T MR system (Discovery MR750, General Electric, Milwaukee, WI, USA) was used for acquiring high‐resolution 3D T1‐weighted and resting‐state fMRI data. Foam padding was employed to minimize head movement, and earplugs were utilized to reduce the noise generated by the scanner. Throughout the data acquisition, participants were instructed to maintain stillness and avoid any motion. Sagittal 3D T1‐weighted images were acquired using a BRAVO sequence with the following parameters: repetition time (TR) = 8.2 ms, echo time (TE) = 3.2 ms, inversion time (TI) = 450 ms, flip angle (FA) = 12°, field of view (FOV) = 256 mm × 256 mm, matrix size = 256 × 256, slice thickness = 1 mm with no gap, and 188 sagittal slices. Resting‐state fMRI data were obtained through a gradient‐echo single‐shot echo planar imaging (GRE‐SS‐EPI) sequence with the parameters below: TR = 2000 ms, TE = 45 ms, FOV = 220 mm × 220 mm, FA = 90°, matrix size = 64 × 64, slice thickness = 4 mm with a 0.5 mm gap, 32 interleaved transverse slices, and a total of 180 volumes.

The resting‐state fMRI data were preprocessed using the Data Processing Assistant for Resting‐State fMRI (DPARSF) toolbox,^27^ which relies on Statistical Parametric Mapping 12 (SPM12, http://www.fil.ion.ucl.ac.uk/spm12). Initially, the first 10 volumes from each participant were excluded to ensure signal equilibrium. The remaining volumes underwent correction for time differences between slices and head motion, with participants exceeding a maximum displacement of 2.0 mm or a maximum rotation of 2.0 degrees being excluded from subsequent analyses. Nuisance covariates, including linear drift, Friston‐24 head motion parameters, global brain signal, white matter signal, cerebrospinal fluid signal, and volumes affected by movement (defined as framewise displacement [FD] exceeding 0.5 mm^28^), were regressed out. To normalize the functional images, each participant's individual structural image was co‐registered with the mean functional image. The transformed structural images were then segmented into gray matter, white matter, and cerebrospinal fluid. Using these segmented images, the normalization parameters from individual native space to the Montreal Neurological Institute (MNI) space were estimated, based on the Diffeomorphic Anatomical Registration Through Exponentiated Lie algebra (DARTEL) algorithm.^29^ Afterwards, the motion‐corrected functional imaging data were normalized to MNI space based on these parameters and resampled to 3‐mm cubic voxels. Finally, a temporal bandpass filter (0.01–0.08 Hz) was applied to reduce the impact of low‐frequency drift and high‐frequency noise.

The ReHo metric was computed following prior studies^6^, ^30^: Kendall's coefficient of concordance (KCC) was employed to calculate ReHo for a given voxel using the time series of that voxel and its 26 nearest neighbors. The resulting KCC value was then assigned to the original voxel, and this procedure was repeated for all other voxels, generating individual ReHo maps. For the purpose of standardization, the ReHo value for each voxel was *z*‐score standardized by subtracting the global mean and dividing by the standard deviation of all ReHo values.

To investigate the impact of global mean signal on the results, we also compared the results between with and without global mean signal regression.

### 2.4 Spatial scale definition

In the present study, a hierarchical approach was employed to define four distinct brain parcellation scales using brain atlases based on anatomical features of the brain. First, Scale 1 was defined at the voxel level, encompassing a total of 67,541 voxels (Gray matter voxels). Afterwards, the entire brain was partitioned into 246 cerebral regions using the Human Brainnetome Atlas (http://atlas.brainnetome.org),^31^ in addition to 26 cerebellum regions based on the anatomical automatic labeling (AAL) atlas.^32^ This partitioning was designated as Scale 2, encompassing a total of 272 regions. Building upon Scale 2, smaller regions were merged into larger ones based on their anatomical associations, resulting in the creation of Scale 3 (consisting of 53 regions) and Scale 4 (comprising 17 regions). For instance, within Scale 2, seven separate subregions existed within the left superior frontal gyrus. When merging Scale 2 into Scale 3 and further into Scale 4, for the Human Brainnetome Atlas template, the merging principle is based on the original subdivisions of the Human Brainnetome Atlas.^31^ For example, these subregions were merged into a single region named the ‘left superior frontal gyrus’ in Scale 3, and this ‘left superior frontal gyrus’ region was further combined with the other six regions in the left frontal lobe to form a unified region known as the ‘left frontal lobe’ in Scale 4. For the 26 cerebellum regions merging method, the merging principle is based on the original subdivisions of the cerebellum of AAL template. For a detailed explanation of Scales 3 and 4, please refer to Table S1.

### 2.5 Classification analysis

To distinguish between schizophrenia patients and HCs, MVPA analysis was conducted, a technique capable of uncovering subtle spatial discriminative patterns and effectively exploring complex, high‐dimensional neuroimaging data.^26^, ^33^, ^34^ Utilizing these four spatial scales, ReHo metrics were obtained at both the voxel level and region level (i.e., mean ReHo value within each region). The ReHo metric at each scale was employed as classification features. To enhance computational efficiency and classification performance by reducing the number of features, the *F*‐score method was applied for feature selection. The *F*‐score of a feature was derived from an F test performed between the patients and the HCs of the training data and thus quantified its discriminative power between the two groups.^26^, ^35^ The features were ranked from high to low according to their *F* scores and then the features with top *F*‐scores were selected using a series of thresholds. Specifically, 10 sets of features (ranging from 1% to 100% of the total number of features, with the step size of 1%) were selected to train the SVMs, resulting in 10 trained models. Subsequently, we tested these 10 models using the test set and the results of the model with the best performance were reported. During training, the SVM kernel parameters was optimized using a cross‐validation framework and was independent of the test data. Additionally, nu‐SVC and C‐SVC, with various kernel functions (including linear, polynomial, and radial basis function) were trained and tested as the classifier, and the kernel parameters (the penalty coefficient C or nu) were optimized using a grid search based on the training dataset: the penalty coefficient C varied from 1 to 100 with a step size of 5, and the parameter nu varied from 0.2 to 0.7 with a step size of 0.1. Therefore, two types of penalty parameters (20 values for parameter C and six values for parameter nu) and three types of kernels were searched during the grid search procedure. Our assessment of the classifier's performance involved a 10‐fold cross‐validation approach,^36^, ^37^ where the dataset was divided into 10 subsets according to the subjects' ID. In each iteration, nine of these subsets were selected for training, while the remaining subset was used for testing. This process was repeated 10 times, with each subset taking a turn as the test set in separate iterations. Classification accuracy served as the measure of the classifier's performance. Subsequently, the statistical significance of classification accuracies was assessed using a nonparametric permutation test, wherein patient and control labels were randomly reassigned, and the same feature selection and classification procedures mentioned above were applied to generate chance‐level classification accuracy. This entire process was repeated 5000 times to establish a null distribution based on these chance‐level classification accuracies. A classification result was considered significant if the actual accuracy, without permutations, was exceeded by fewer than 5% of all permutations, indicating that the classification accuracy was unlikely to occur by chance. The entire MVPA analysis was implemented using the MVPANI package.^26^

We further identified the features contributing to the classifications at Scale 1 and Scale 2 according to feature weights: the features with top 20% absolute weight values among the features selected across all 10 cross‐validation steps were considered to make important contributions to the classifications. Note that, this analysis was only performed for Scale 1 and Scale 2 because the support vector machines with non‐linear kernels were used for Scale 3 and Scale 4 and thus feature weights indicating contributions to classifications could not be derived for Scale 3 and Scale 4. The correlations between the ReHo values of these identified important features and each of the PANSS scores (total, positive, negative, and general scores) were explored using the Pearson's correlation analysis (false discovery rate [FDR]‐corrected *p* < 0.05).

### 2.6 Transcriptome‐neuroimaging association analysis

Publicly available, normalized microarray expression data from the AHBA database were acquired.^38^ Among the six donors, only two had expression data available for both hemispheres, while the remaining four donors had data for the left hemisphere. Therefore, our analysis focused exclusively on the left hemisphere of these six donors. A processing pipeline, whose code is available on GitHub (https://github.com/BMHLab/AHBAprocessing) with detailed parameter configurations listed in Table S2, was employed to link whole‐brain gene expression profiles to neuroimaging data.^24^ Initially, probes were reassigned to genes using the latest National Center for Biotechnology Information (NCBI) database. Then, probes with expression intensities below the background signal in over 50% of samples were excluded. Afterwards, the genes that have no corresponding RNA‐seq measures were removed. Following this, the probes that had low correlations with RNA‐seq data (Spearman rho <0.2) were excluded. Finally, a representative probe for a gene based on the highest correlation to RNA‐seq gene expression data in corresponding samples was selected. Differential stability (DS), a correlation‐based metric, was applied to assess the reliability of expression patterns in differentially expressed genes across brain structures in the six donor brains.^39^ Genes were ranked based on their DS values, and the top half of high‐DS genes were selected for correlation analysis with neuroimaging data. With this pipeline, a gene expression matrix for Scale 1 was obtained, with dimensions of 5093 genes × 1782 samples. When performing the transcriptome‐neuroimaging association analysis in region‐level Scales 2–4, the allocation of samples to specific regions within the respective parcellation schemes was determined based on the closest Euclidean distance, and the expression values of all samples within each region were averaged, providing the expression levels for each brain region. The dimensions of expression matrices for Scale 2, Scale 3, and Scale 4 were 5093 genes × 138 regions, 5093 genes × 27 regions, and 5093 genes × 9 regions, respectively.

WGCNA, a powerful bioinformatics method extensively used in genomics and systems biology, was utilized to categorize genes into network modules and reveal biologically significant insights.^40^ At each chosen spatial scale, a signed network is established using the gene × sample/region expression matrix, wherein the strength of co‐expression relationships is determined through a soft thresholding technique. Next, hierarchical clustering is employed to identify modules consisting of co‐expressed genes. The expression pattern for each module, represented as a matrix (number of samples × number of genes within the module), is subsequently condensed into its first principal component, known as the module eigengene (ME). This ME is represented as a vector with dimensions of number of samples × 1, providing a summary of the module's overall expression profile.

ReHo difference maps were generated by comparing schizophrenia patients with HCs at both the voxel level (Scale 1) and regional level (Scales 2–4) using two‐sample *t*‐tests or non‐parametric test according to whether the corresponding ReHo data were normally distributed, while controlling for age and gender as nuisance covariates. Kolmogorov–Smirnov tests were employed to test the normality of ReHo values at regional level (Scales 2–4). For the comparisons of ReHo values at voxel level (Scale 1), the standard procedure using the two‐sample *t*‐tests was performed using the software package SPM12.^41^, ^42^, ^43^ Furthermore, a transcriptome‐neuroimaging association analysis was carried out to explore the relationship between transcriptional profiles and ReHo differences. Specifically, at Scale 1, a sample‐level spatial correlation analysis was performed, where the mean *t*‐value within a 6 mm sphere centered on each tissue sample was extracted from the uncorrected case–control ReHo difference map and correlated with each ME. At Scales 2–3, a regional level spatial correlation analysis was performed, involving the calculation of the mean *t*‐value within each region from the ReHo difference map and its correlation with each ME. Notably, the spatial correlation analysis was separately conducted for subcortical and cortical samples due to significant differences in gene expression profiles between these regions.^38^ At Scale 4, a total of nine sampling points were extracted from the left hemisphere to construct expression matrices, which was insufficient to meet the sample size recommended by the WGCNA website (at least 15 samples). Therefore, WGCNA analysis was not conducted at Scale 4 to ensure the reliability of the study findings.

### 2.7 Enrichment analysis

The genes within significant modules were aggregated at each scale, and enrichment analysis related to gene ontology (GO) was performed on these genes using the Toppogene (https://toppgene.cchmc.org/) to identify significant enrichments. In this analysis, all enrichment analyses were corrected by BH‐FDR *p* < 0.05.

## 3 RESULTS

### 3.1 Demographic and clinical characteristics of participants

The demographics and clinical characteristics of the patients with schizophrenia are presented in Table 1. A total of 213 right‐handed participants were recruited for this study, which included 103 patients with schizophrenia. The mean age for the patients with schizophrenia was 33.9 years (±9.6), and the group consisted of 49 females and 54 males. Additionally, 110 healthy controls were included in the study, with a mean age of 33.7 years (±11.0), and the group comprised 65 females and 45 males. There were no significant differences in age and gender distribution between the two groups, and no participants were excluded from the study due to excessive head motion.

**TABLE 1:** Demographic and clinical characteristics of participants.

	Schizophrenia	HCs	*p* Value
Sample size	103	110	
Illness duration (months)	116.6 ± 95.9	–	
Age (years)	33.9 ± 9.6	33.7 ± 11.0	0.856^a^
Gender (M/F)	54/49	45/65	0.092^b^
Handedness (R/L/B)	103/0/0	110/0/0	
PANSS total score	71.1 ± 22.3	–	
PANSS positive score	16.8 ± 7.7	–	
PANSS negative score	20.0 ± 8.9	–	
PANSS general score	34.3 ± 10.5	–	

Abbreviations: HCs, healthy controls; PANSS, Positive and Negative Syndrome Scale.

^a^ Two‐sample *t*‐test.

^b^ Chi‐square test.

### 3.2 Classification analysis utilizing ReHo metric across four spatial scales

The classification accuracies of the ReHo metric for distinguishing schizophrenia from HCs across four spatial scales are presented in Figure S1. Our findings revealed that the accuracy of ReHo classification increased from 83.1% at Scale 1%–84.6% at Scale 2. However, this accuracy gradually decreased from 78.5% at Scale 3%–74.2% at Scale 4, as the spatial scale expanded. Importantly, all of these results significantly exceed chance levels (*p* < 0.001), highlighting the stability of ReHo as a neuroimaging biomarker for schizophrenia classification. Detailed information on the optimal parameters obtained through grid search for achieving the highest classification accuracy in the four scales is shown in Table S3. The features with consistently high weights (top 20% absolute weight values) for Scale 1 and Scale 2 are shown in Figure S2. The overlapping regions between Scale 1 and Scale 2 were indicated in red in Figure S2C. These indicated that there were some consistent features (brain regions) in both Scale 1 and Scale 2, furthermore, all the consistent features (brain regions) can be observed in Scale 2. The detailed information of brain regions were shown in Table S4.

Among the brain regions with high weight (Table S4), the ReHo values of FuG_R_2 was positively correlated with PANSS negative score (correlation coefficient = 0.32; *p* = 0.001; Figure S3A); ReHo values of Hipp_L_2 was negatively correlated with PANSS general scores (correlation coefficient = −0.29, *p* = 0.003; Figure S3B) and PANSS total score (correlation coefficient = −0.31, *p* = 0.002; Figure S3C). No other significant correlations were found between ReHo values and PANSS scores.

The results obtained without global signal regression showed that the performance of the classification model was decreased compared with those obtained with the global signal regression (Tables S3 and S5). This decrease in performance might be attributed to the presence of global noise and non‐neuronal fluctuations within the global mean signal, which would obscure the true neural activity patterns and reduce the signal‐to‐noise ratio.

### 3.3 Case–control difference in ReHo metric across four spatial scales

After controlling for age and sex, ReHo values at regional level (Scales 2–4) for HCs and patients with schizophrenia were normality distributed, confirmed by Kolmogorov–Smirnov tests (Table S6), and thus two‐sample *t*‐tests were employed to identify differences in ReHo between patients and HCs for each scale. The results showed that, compared with HCs, the patients with schizophrenia showed altered ReHo in multiple brain regions at each scale (Figure S4; Scale 1: *p* < 0.05, FWE corrected; Scale 2: *p* < 0.05/272 = 1.83 × 10^−4^; Scale 3: *p* < 0.05/54 = 9.3 × 10^−4^; Scale 4: *p* < 0.05/17 = ×0.0029; all Bonferroni‐corrected).

### 3.4 Transcriptome‐neuroimaging association analysis

**FIGURE 2:**
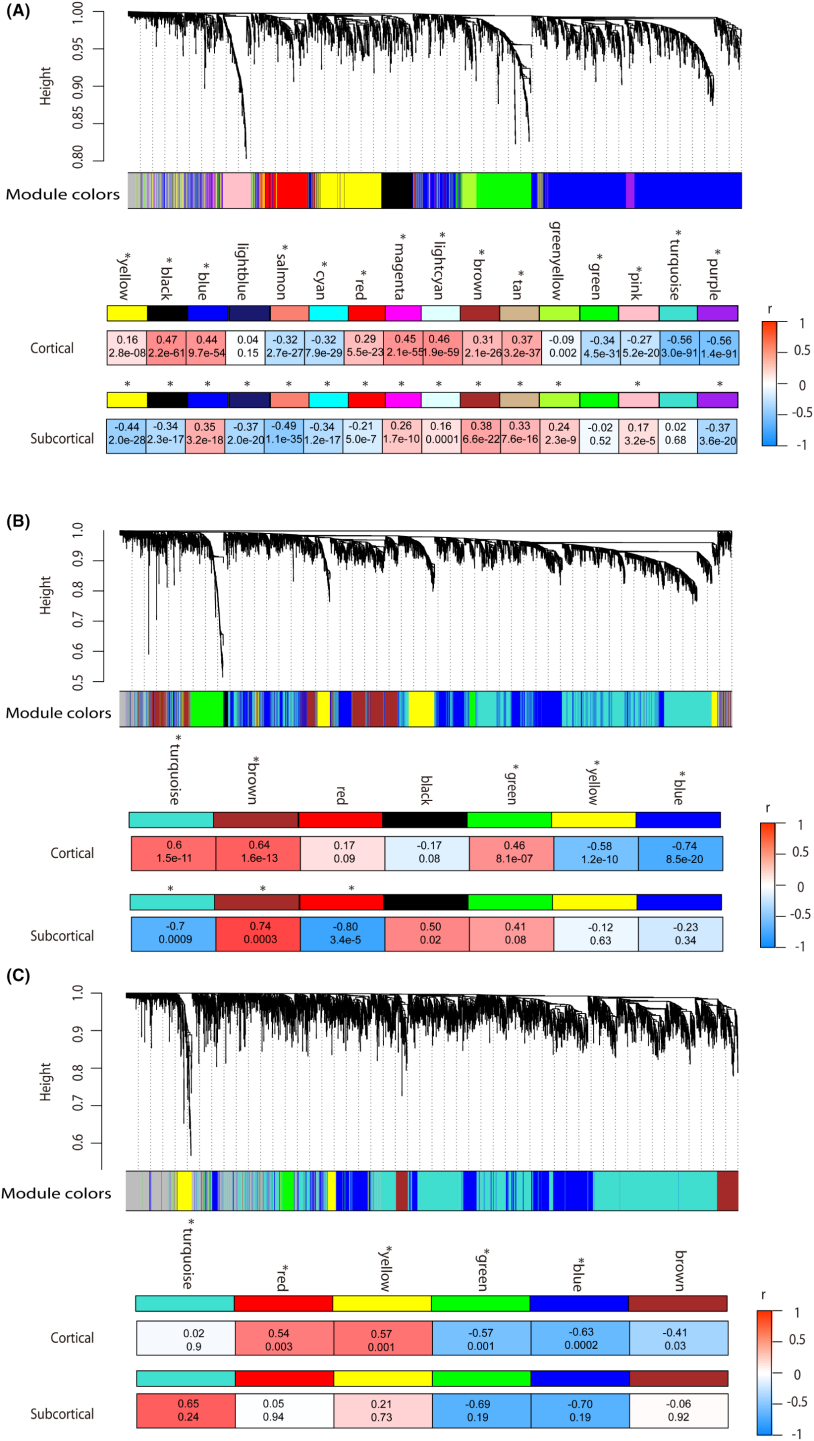
Pearson's correlation between MEs of gene modules and *t*‐statistics values of case–control ReHo differences in cortical and subcortical regions of schizophrenia at Scales 1–3. (A–C) Showed the correlation coefficients between MEs of gene modules and ReHo differences in Scale 1 (voxel level), Scale 2 (272 cerebral regions), and Scale 3 (53 regions), respectively. The color bar represents the correlation coefficients. At Scale 4 (17 regions), the limited number of cerebral regions prevented the implementation of WGCNA analysis, leading to underpowered analysis and inaccurate network modules.

The expression pattern for each module was shown in Table 2. At Scale 1 (voxel‐level), WGCNA analysis identified 17 distinct modules. Subsequent spatial correlation analysis at the sample level demonstrated significant associations with 14 of these modules in both cortical and subcortical regions (Figure 2A). When examining Scale 2 (272 regions), we found a total of eight modules. Among them, five were significantly associated with cortical regions, while three modules showed significance in subcortical regions (Figure 2B). At Scale 3 (53 regions), we identified seven modules. Of these, four were significant in cortical regions, while none exhibited significance in subcortical regions (Figure 2C).

**TABLE 2:** The number of genes in each module.

Module	Number of genes	Module	Number of genes
Scale 1			
Black	153	Midnightblue	44
Blue	1163	Pink	125
Brown	427	Purple	99
Cyan	45	Red	190
Green	261	Salmon	45
Greenyellow	77	Tan	75
Gray	708	Turquoise	1202
Lightcyan	41	Yellow	330
Magenta	108	All	5093
Scale 2			
Black	38	Gray	328
Blue	1251	Red	149
Brown	744	Turquoise	1877
Green	324	Yellow	382
		All	5093
Scale 3			
Blue	1039	Red	74
Brown	254	Turquoise	2124
Green	135	Yellow	183
Gray	1284	All	5093

### 3.5 Enrichment analysis

GO enrichment analysis was performed for the genes associated with ReHo differences. Detailed results, including enriched biological processes, molecular functions, and cellular components, can be found in Table S7.

**FIGURE 3:**
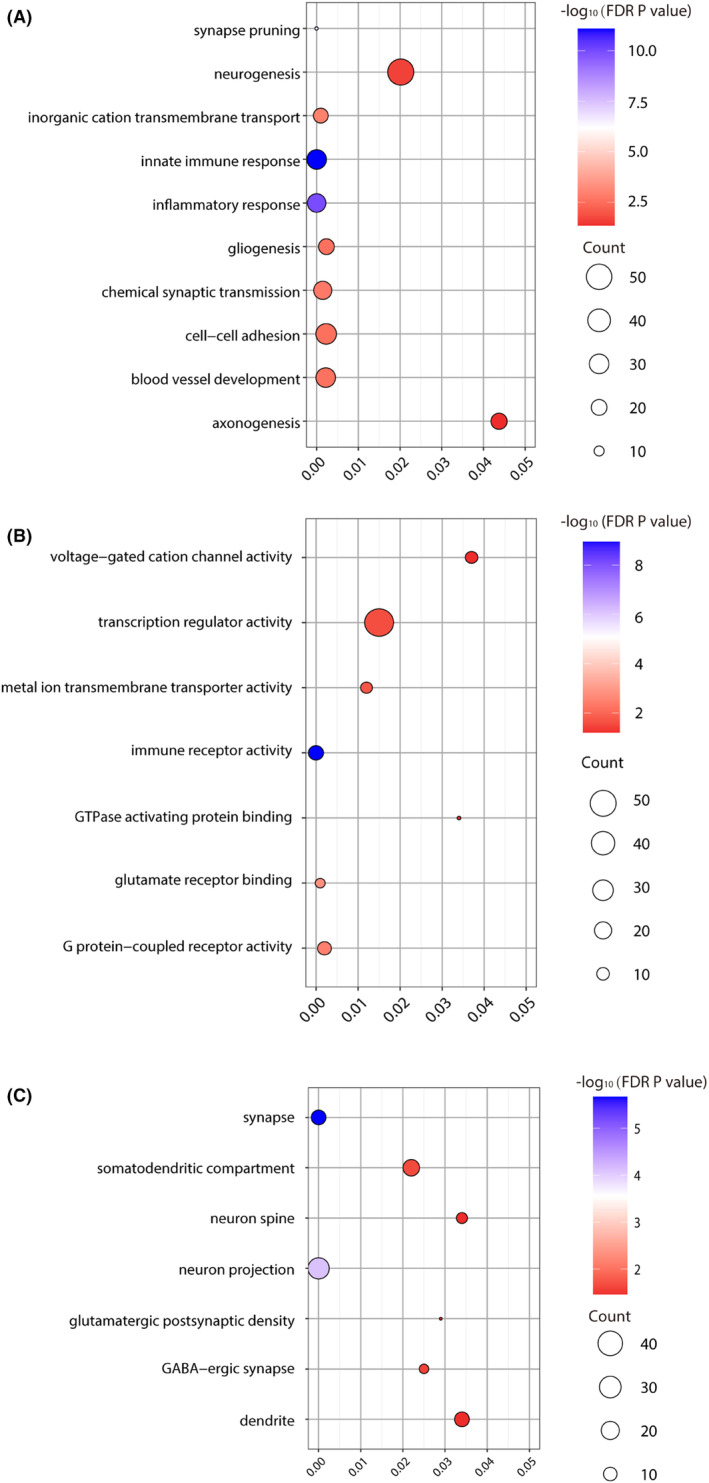
Gene enrichment of genes significantly correlated with ReHo alterations in schizophrenia in Scale 1. (A) Significant GO items of biological processes; (B) Significant gene ontology (GO) items of molecular function; (C) Significant GO items of cellular components. The x‐axis represented the *p* value of enrichment for each GO item (*y*‐axis). The size of each sphere indicated the number of genes overlapped with each GO item, and the color of each sphere indicated the significance level of enrichment, as shown in the color bar.

In terms of biological processes, in Scale 1, genes were enriched for pathways related to innate immune response, inflammatory response, synapse pruning, chemical synaptic transmission, inorganic cation transmembrane transport, cell adhesion, blood vessel development, gliogenesis, neurogenesis, and axonogenesis (Figure 3A). In Scale 2, in addition to the pathways identified in Scale 1, genes were also enriched for pathways related to ion transmembrane transport, cell activation/migration, and the G protein‐coupled receptor signaling pathway (Figure S5B). In Scale 3, genes were enriched for pathways related to myelination, axon ensheathment/regeneration, cell–cell signaling, neuron development, and signal release (Figure S6B).

For molecular functions, in Scale 1, genes in these significant modules were enriched for pathways related to immune receptor activity, G protein‐coupled receptor activity, voltage‐gated cation channel activity, glutamate receptor binding, metal ion transmembrane transporter activity, GTPase‐activating protein binding, and transcription regulator activity (Figure 3B). In Scale 2, apart from the pathways identified in Scale 1, genes were also enriched for pathways related to GABA receptor activity, amino acid transmembrane transporter activity, and molecular transducer activity (Figure S5A). In Scale 3, genes were enriched for pathways related to transporter activity, transmembrane transporter activity, calcium ion transmembrane transporter, and estrogen receptor activity (Figure S6A). In terms of cellular components, in Scale 1, genes were enriched for pathways related to synapse, dendrite, neuron spine/projection, GABAergic synapse, somatodendritic compartment, and glutamatergic synapse (Figure 3C). In Scale 2, in addition to the pathways identified in Scale 1, genes were also enriched for pathways related to dopaminergic synapse, cholinergic synapse, and GABA receptor complex (Figure S5C). In Scale 3, genes were enriched for pathways related to axon, synaptic vesicle, cell body, hippocampal mossy fiber to CA3 synapse, and myelin sheath (Figure S6C).

## 4 DISCUSSION

To our knowledge, this study represents the first attempt to explore the diagnostic potential of multi‐scale ReHo and its molecular foundations in schizophrenia. Specifically, a comprehensive examination spanning various spatial scales, encompassing fine‐grained voxel‐level and broader region‐level parcellations, is provided, thereby highlighting the robustness of ReHo as a reliable neuroimaging biomarker for schizophrenia classification. Additionally, an extensive transcriptome‐neuroimaging association analysis is included, unveiling compelling connections between multi‐scale ReHo differences and genes associated with immune responses, receptor activities, and synaptic components.

A significant classification accuracy for schizophrenia was achieved at both fine‐grained voxel‐level and coarser region‐level scales. Scale 2, composed of 272 regions, achieved the highest classification accuracy at 84.6%, while Scale 1 (voxel‐level) exhibited an accuracy of 83.1%. Conversely, Scale 4, comprising 17 regions, demonstrated the lowest accuracy at 74.2%. The differences in classification accuracy among different spatial scales could be due to the trade‐off between information loss and signal‐to‐noise ratio (SNR) improvement. The Scale 2 exhibited the highest classification accuracy compared to the other scales, likely due to an optimal trade‐off: a relatively small information loss and a considerable gain of SNR compared to the Scale 1. As the spatial scale expands from 272 regions to 17 regions, classification accuracy gradually decreased, suggesting an increasing information loss. This emphasizes that, with an increase of spatial scale (i.e., a decrease in region number), the impact of information loss becomes more prominent than the SNR improvement, resulting in a decreased classification accuracy. At Scale 2, brain regions such as the bilaterally cerebellum, inferior frontal gyrus, superior temporal gyrus, inferior temporal gyrus, fusiform gyrus, postcentral gyrus, thalamus, and insular gyrus were found to contribute significantly to the classification (i.e., showing high weights). The structural or functional abnormalities in these brain regions have been reported in schizophrenia.^44^, ^45^, ^46^ The ranges of classification accuracy in previous studies using ReHo were from 72.49% to 90.14%.^9^, ^30^, ^47^, ^48^ The important features contributing to the classifications were consistent with those of our findings.^9^, ^30^ Furthermore, our results showed that the ReHo metrics could successfully classify the patients with schizophrenia from HCs at each Scale, indicating that ReHo has the potential to serve as a diagnostic tool in clinical settings. Considering that current schizophrenia diagnosis mainly relies on subjective evaluation, such objective neuroimaging biomarkers could complement the traditional subjective method and enhance the diagnosis precision.

In the transcriptome‐neuroimaging association analysis, we found that the number of gene modules decreased with the increase of the spatial scale: the largest number of gene modules (17 modules) was identified for Scale 1 and the least (eight modules) were identified for Scale 3. One possible reason is the information loss caused by merging voxels/smaller regions into larger regions from Scale 1 to Scale 3, and thus less biological pathways/functions could be identified through the transcriptome‐neuroimaging association analysis. Similarly, Scale 1 exhibited the highest number of biological pathways. Therefore, our findings suggest that Scale 1, with the highest number of biological pathways and relatively high classification accuracy, provides the richest information. Scale 2, with gene modules similar to Scale 1, exhibits slightly higher classification accuracy. On the other hand, Scale 3 has fewer biological pathways and lower classification accuracy, may be associated with its specific gene modules. Besides the number of biological pathways, the unique GOs in Scale 1 included the ribonucleoprotein complex, glutamatergic postsynaptic density, GABA‐ergic synapse, postsynaptic density membrane, parallel fiber to Purkinje cell synapse, voltage‐gated potassium channel complex, and adherens junction, etc. The unique GOs in Scale 2 included dopaminergic synapse, cholinergic synaps, and GABA receptor complex, and the unique GOs in Scale 3 included hippocampal mossy fiber to CA3 synapse, hippocampal mossy fiber, myelin sheath, and synaptic vesicle. These unique GOs in each scale have been reported to be involved in the etiologies of schizophrenia and might also cause the different classification accuracy.^49^, ^50^, ^51^, ^52^, ^53^, ^54^, ^55^, ^56^, ^57^ Moreover, shared biological pathways were identified within Scales 1–3, as well as between any two of these scales. Notably, the number of shared gene profiles between Scales 1 and 2 significantly exceeded that between Scales 1 and 3. These results offer a potential explanation for the subtle variation in classification accuracy between Scales 1 and 2 in the context of schizophrenia, while revealing a more pronounced difference in classification accuracy between Scales 1 and 3. Furthermore, distinct biological pathways were evident within each individual scale, serving as an indication that different scales hold unique information, alongside the shared elements. The presence of shared and specific gene modules across different scales highlights the complex nature of the molecular basis of schizophrenia.

Our enrichment analysis revealed that genes associated with ReHo alterations in schizophrenia were significantly enriched in pathways related to the innate immune response, inflammatory response, and microglial cell activation. These findings align with previous research indicating that immune dysregulation and inflammation play crucial roles in the pathophysiology of schizophrenia.^58^, ^59^ For instance, increased levels of pro‐inflammatory cytokines have been observed in patients with schizophrenia, suggesting that inflammation may contribute to neural abnormalities and cognitive impairments seen in this disorder.^60^ Additionally, we found significant enrichment in pathways involved in synaptic signaling, chemical synaptic transmission, and neurotransmitter regulation, including glutamatergic and GABAergic signaling. These pathways are critical for maintaining normal synaptic function and neural communication. Dysregulation in these signaling pathways has been implicated in schizophrenia, contributing to the synaptic deficits and cognitive dysfunctions characteristic of the disorder.^61^ For example, alterations in glutamate signaling have been associated with both positive and negative symptoms of schizophrenia.^62^ Furthermore, pathways related to ion transmembrane transport, voltage‐gated ion channel activity, and neurodevelopmental processes such as axonogenesis and gliogenesis were also significantly enriched. Abnormal ion channel functioning can affect neuronal excitability and synaptic transmission, leading to the neurophysiological abnormalities observed in schizophrenia.^63^ Furthermore, disruptions in neurodevelopmental processes during critical periods of brain development may underlie the structural and functional brain abnormalities seen in patients. A recent study by Ji and colleagues also found these biological processes in connection with alterations in gray matter volume in schizophrenia.^43^ These findings imply a relationship between these biological processes and changes in neuroimaging phenotypes, including alterations in cerebral functional activity and structural characteristics, among individuals with schizophrenia. Moreover, several biological pathways were common among Scales 1–3, encompassing synaptic processes such as synaptic signaling, chemical synaptic transmission, glutamatergic synapse, pre‐ and post‐synapse functions, as well as signal release, neuron projection, dendrite, cell surface, and ion transmembrane transport activity, suggesting that potential fundamental mechanisms for schizophrenia could involve neuronal dysfunction and developmental irregularities.^64^ Furthermore, biological pathways related to cellular components (e.g., glutamatergic synapse, synapse, pre‐ and post‐synapse, dendrite, neuron projection), and molecular functions (e.g., voltage‐gated cation and ion channel activity) have also been previously linked to the onset of schizophrenia.^65^

The shared biological pathways between Scales 1 and 2 comprise a range of critical processes, including microglial cell activation, glial cell development and migration, innate and adaptive immune responses, inflammatory reactions, G protein‐coupled receptor signaling pathways, retinoid metabolic processes, and blood vessel development and morphogenesis. Disruption in genes responsible for functions such as cell adhesion, migration, proliferation, synaptic transmission, signal transduction, and glial development has been shown to have implications for brain development and can contribute to the onset of schizophrenia.^43^, ^65^ In accordance with this, prior studies suggest that genetics, transcriptomics, post‐mortem analysis, epidemiology, peripheral biomarkers, and therapeutic interventions for schizophrenia collectively indicate dysregulation in both adaptive and innate immune systems, actively influencing the condition's symptoms.^66^, ^67^ Microglial cells, being central immune cells of the central nervous system, play a pivotal role in inflammatory responses, neuronal remodeling, and synaptic pruning.^68^ Additionally, we observed correlations between the ReHo biomarker and gene modules enriched for blood vessel development and morphogenesis, which may be attributed to the ReHo's foundation in the neurovascular coupling theory of blood oxygen level dependent (BOLD) signals, where brain vessels are integral components of the neurovascular unit.^69^ These findings align with previous studies, such as the work of Xue et al.,^64^ reinforcing our hypotheses. Furthermore, our study identified correlations between ReHo and gene modules enriched for the retinoid metabolic process, which is intrinsically linked to neural development, connectivity, plasticity, and the pathophysiology of schizophrenia, particularly in patients with severe cognitive impairment.^70^ The retinoid pathway has been implicated in synaptic plasticity, thereby influencing brain function and behavior. Collectively, these results emphasize the pivotal role these pathways play in brain function and the development of schizophrenia.

In Scale 3, a significant correlation was found between the ReHo and the expression profiles of gene modules enriched for myelination processes, with a particular focus on central nervous system myelination and axon ensheathment in the central nervous system. Myelination plays a pivotal role in shaping neural circuit plasticity, a fundamental aspect of brain function that governs precise timing and overall brain performance.^71^ It is essential to recognize that myelination represents an evolutionary advancement critical for sensory, motor, and higher‐order cognitive functions. Myelin, a complex multilayered structure derived from the oligodendrocyte plasma membrane, functions to wrap axons, enabling efficient electrical conduction.^72^ Disruptions in glial function, glial structure, or glial‐neuronal interactions have been linked to myelin deficits in various psychiatric disorders, underlining the importance of these findings in understanding neurological conditions.^73^ All these insights gained from the transcriptome‐neuroimaging association analysis not only provide a deeper understanding of the molecular underpinnings of the ReHo alterations in schizophrenia but also lay the foundation for more accurate diagnosis and effective individualized treatment of schizophrenia based on one's genetic profile.

Our study has several limitations. First, in our transcription‐neuroimaging association analysis, we did not integrate gene expression and neuroimaging data from the same individuals. As a result, our analysis focused solely on genes that showed consistent expression patterns across various brain regions in different subjects. Second, the sample size for individuals with schizophrenia was relatively small and the patients were medicated chronic patients with various symptom severity. These factors might have affected the observed ReHo changes, and thus future studies with a larger group of first‐episode, drug‐free schizophrenia patients are needed to validate our results. Third, an anatomical atlas was used to generate different spatial scales of brain parcellations in the present study; however, functional parcellations (e.g., Schaefer's parcellation) might be more appropriate for investigating the alterations of brain functional metrics (e.g., ReHo), which will be tested in future studies. Fourth, in this study, we only tested F‐scores for feature selection, and other approaches such as Lasso regularization or PCA might perform better and will be tested in future studies. Finally, the gene expression data used in this study are not specific to East Asian populations, as they originate from the AHBA, which is currently the only source for high‐resolution gene expression data. Since there is no available East Asian gene expression dataset at present, future research aiming to replicate our findings should consider using gene expression profiles from Asian brains.

## 5 CONCLUSION

In summary, the multi‐scale ReHo analysis has demonstrated its ability as a neuroimaging biomarker for distinguishing individuals with schizophrenia from HCs. Notably, Scale 2, which comprises 272 regions, has shown the highest classification accuracy. Our exploration of the relationship between ReHo alterations and genes associated with immune responses, synaptic functions, and receptor activities, through transcriptome‐neuroimaging analysis, has revealed significant connections. The examination of diverse spatial scales has uncovered both shared and unique biological pathways contributing to ReHo changes in schizophrenia, emphasizing the intricate nature of the disorder. Overall, this study not only improves diagnostic accuracy but also provides valuable insights into the molecular underpinnings of the condition, paving the way for future advancements in diagnosis and personalized medicine.

## AUTHOR CONTRIBUTIONS

Meng Liang designed the study and revised the manuscript. Feng Liu revised the manuscript. Yanmin Peng and Chao Chai wrote the paper. Yanmin Peng, Kaizhong Xue, Jie Tang, Sijia Wang, and Qian Su analyzed data. Chongjian Liao, Guoshu Zhao, Shaoying Wang, Nannan Zhang, Zhihui Zhang, and Minghuan Lei, contributed to the data interpretation. All authors contributed to and have approved the final manuscript.

## CONFLICT OF INTEREST STATEMENT

The authors have no conflict of interest to declare.

## Supporting information


Figures S1–S6

Tables S1–S7

## Data Availability

The data that support the findings of this study are available from the first author or the corresponding author upon reasonable request.
