# Supplementary material for: Unraveling multi‐scale neuroimaging biomarkers and molecular foundations for schizophrenia: A combined multivariate pattern analysis and transcriptome‐neuroimaging association study
Source: CNS Neurosci Ther. 2024 Aug 8;30(8):e14906. doi: 10.1111/cns.14906 (PMC11310100; doi:10.1111/cns.14906)
Supplement: Supplementary file 1 — Figures S1–S6 [file CNS-30-e14906-s002.docx]

**Supplementary Table 1. The cerebral regions in Scales 1-4**

**Supplementary Table 2. The detailed options for processing gene expression data code**

**Supplementary Table 3. The best parameters of ReHo biomarker for the highest classification accuracy in each spatial scale**

**Supplementary Table 4. Brain regions with the high weight that make significant contributions to discrimination between schizophrenia patients and healthy controls in scale 2 using MVPA**

**Supplementary Table 5. The performance of the classification model in each spatial scale without regressing the global signal in the preprocessing step**

**Supplementary Table 6. Kolmogorov-Smirnov test results for ReHo values at spatial scales 2-4**

**Supplementary Table 7. GO enrichment analysis of each significant module related to the ReHo differences in Scales 1-3**

**Supplementary Figure 1. The classification accuracies of ReHo between schizophrenia and HCs in four spatial scales using MVPA**

**Supplementary Figure 2**. **Weight maps of brain regions that make significant contributions to distinguishing between schizophrenia patients and healthy controls in 2 spatial scales using MVPA**

**Supplementary Figure 3. The correlations between these ReHo values of the brain regions and PANSS scores**

**Supplementary Figure 4. ReHo differences between schizophrenia patients and healthy controls in 4 spatial scales**

**Supplementary Figure 5. Gene enrichment of genes significantly correlated with ReHo alterations in schizophrenia in Scale 2**

**Supplementary Figure 6.** **Gene enrichment of genes significantly associated with ReHo alterations in schizophrenia in Scale 3**


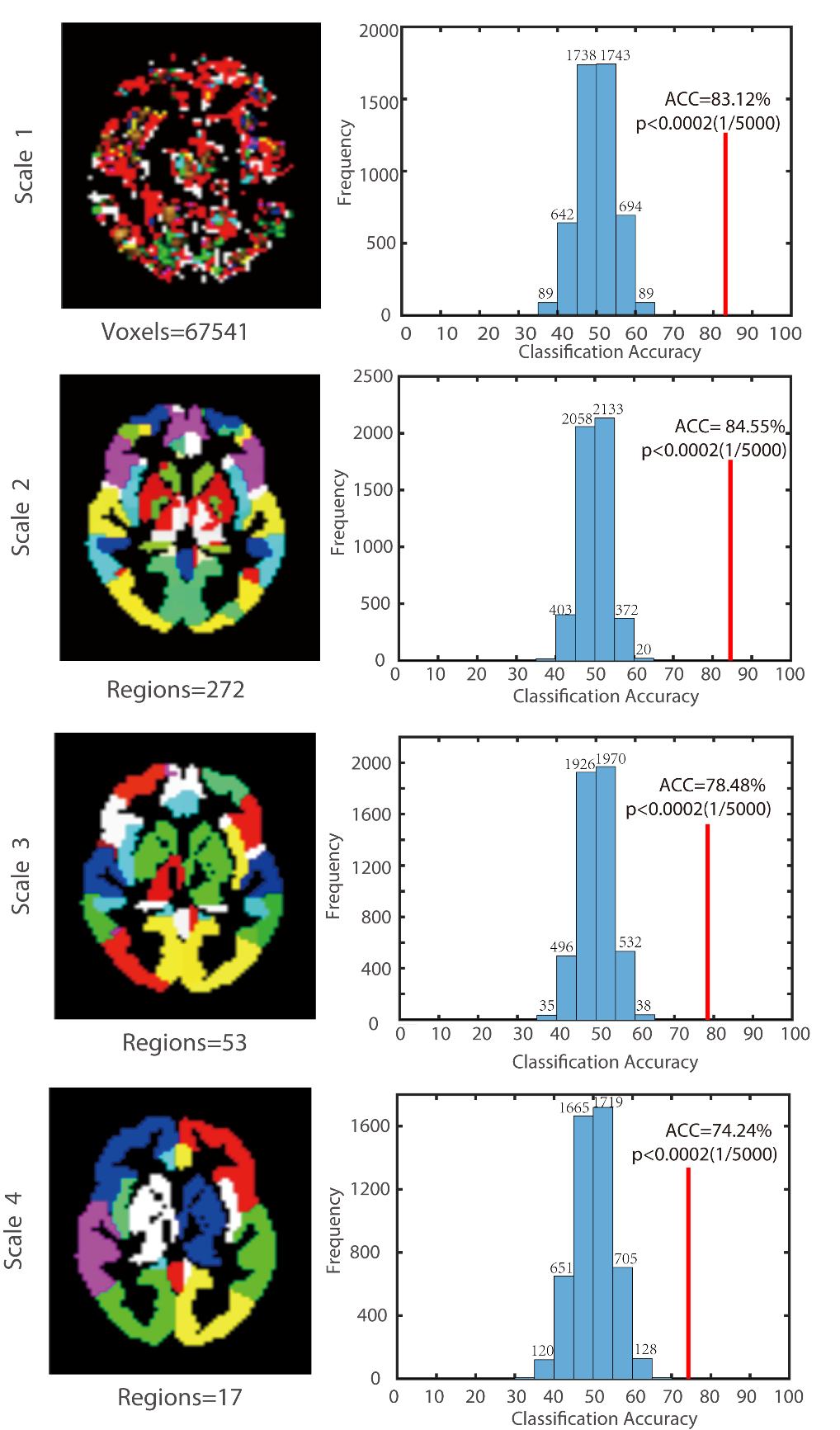


**Supplementary Figure 1**. **The classification accuracies of ReHo between schizophrenia and HCs in four spatial scales using MVPA.** The classification accuracies of ReHo in all spatial scales were significantly distinguishable between patients and HCs. From Scale 1 to Scale 2, the classification accuracies increased from 83.1% to 84.6% and then gradually decreased from 78.5% to 74.2% as the spatial scale became larger when from Scale 3 to Scale 4. 5000 permutations were applied to generate the null distribution. All were significantly higher than the chance level (*P*<0.0002=1/5000).


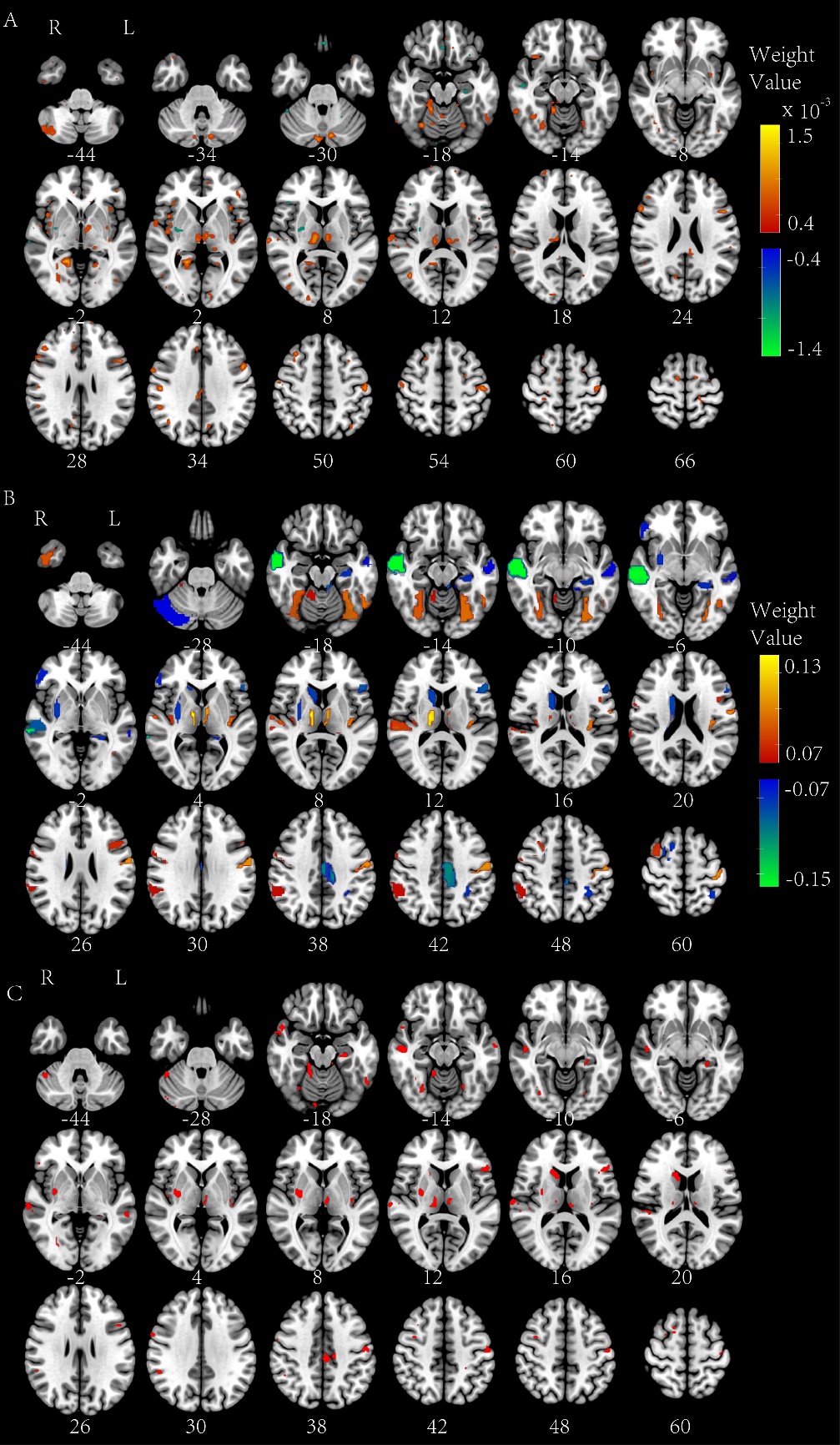


**Supplementary Figure 2**. **Weight maps of brain regions that make significant contributions to discrimination between schizophrenia patients and healthy controls using MVPA at Scales 1 and 2.** Panel A shows the weight map of brain regions that contribute significantly to the classification at Scale 1, while Panel B represents the weight map at Scale 2. The color of each voxel (Panels A and B) indicates the weight (cold color indicates negative weights and warm color indicates positive weights). The red region in panel C represents the overlapping regions between A and B.


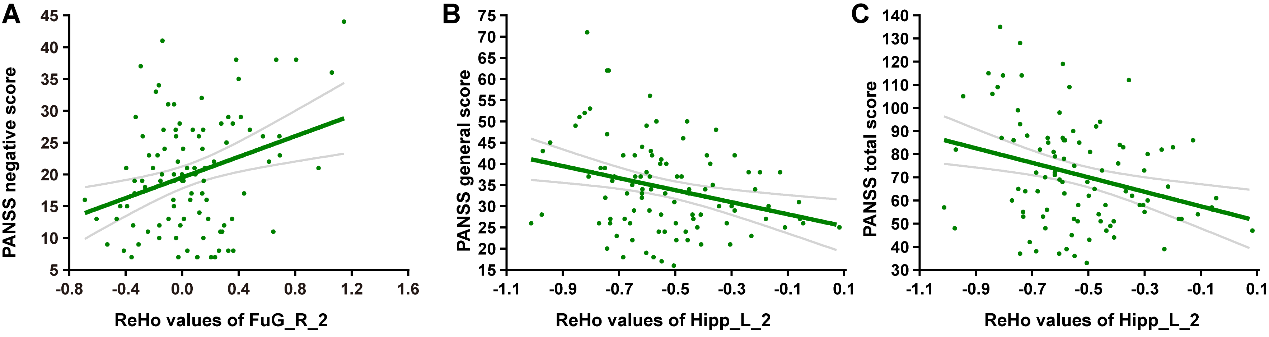


**Supplementary Figure 3**. **The correlations between these ReHo values of the brain regions and PANSS scores.** The brain regions with the high weight that make significant contributions to discrimination between schizophrenia patients and healthy controls in 2 spatial scales using MVPA. Panel A shows the correlations between these ReHo values of the FuG_R_2 and PANSS negative scores. Panel B shows the correlations between these ReHo values of the Hipp_L_2 and PANSS general scores. Panel C shows the correlations between these ReHo values of the Hipp_L_2 and PANSS total scores.


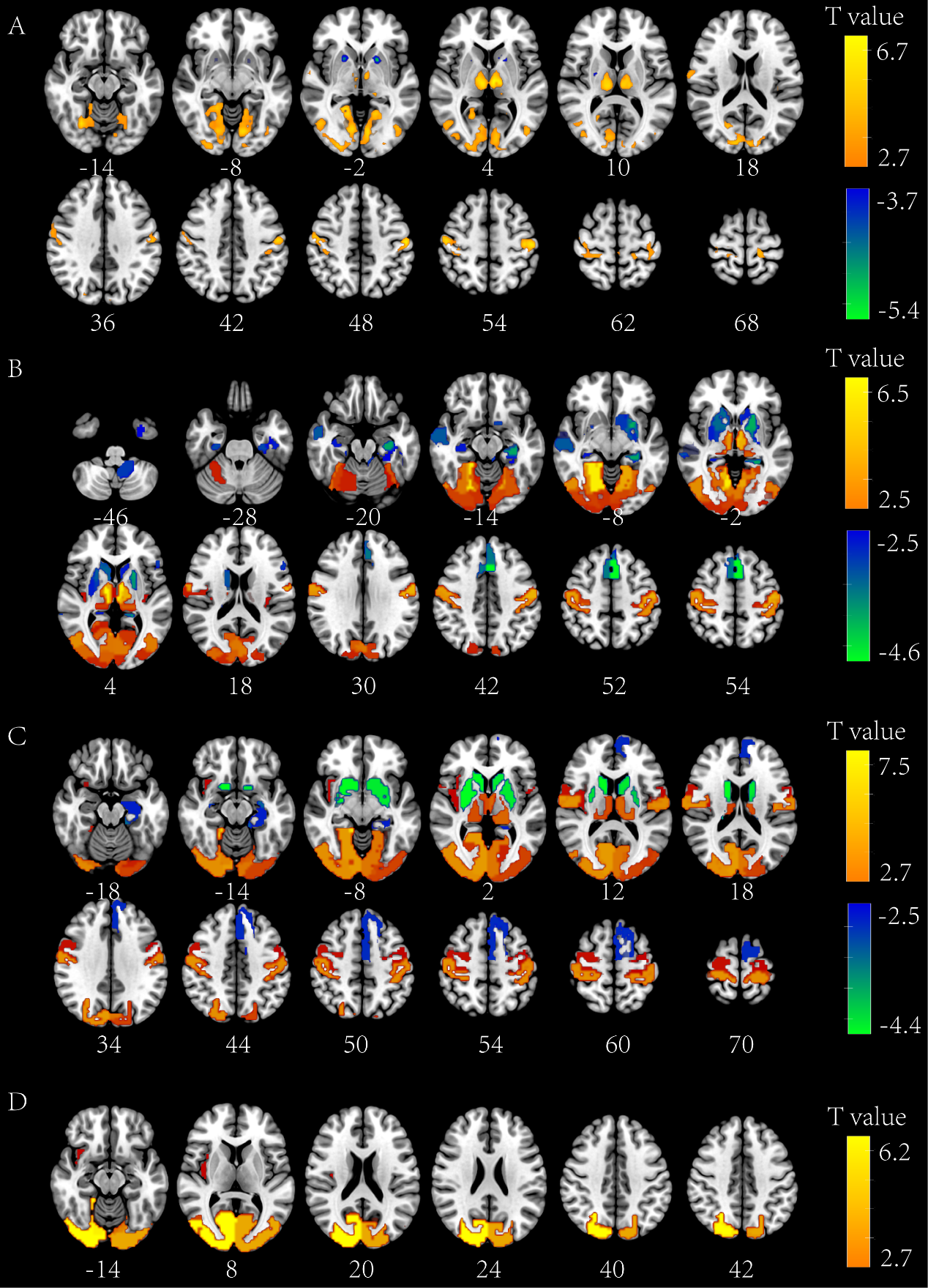


**Supplementary Figure 4**. **ReHo differences between schizophrenia patients and healthy controls in 4 spatial scales (**Scale 1: Panel A; Scale 2: Panel B; Scale 3: Panel C; Scale 4: Panel D). The color bar represents t-values, with positive values indicating higher ReHo in healthy controls than schizophrenia patients, and negative values indicating lower ReHo in healthy controls than schizophrenia patients.


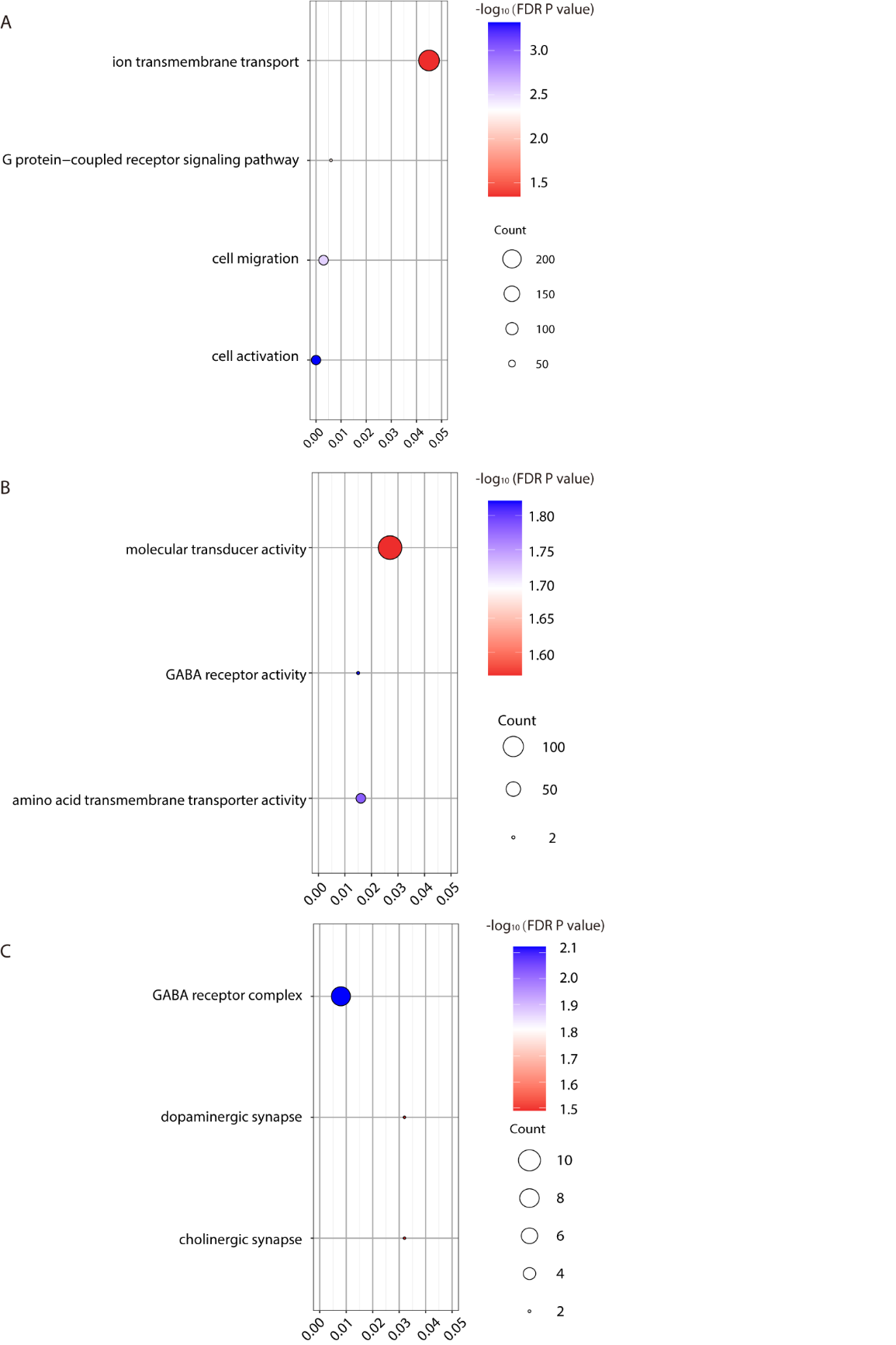


**Supplementary Figure 5. Gene enrichment of genes significantly correlated with ReHo alterations in schizophrenia in Scale 2.** A. Significant gene ontology (GO) items of molecular function; B. Significant GO items of biological processes; C. Significant GO items of cellular components. The x-axis represented the P value of enrichment for each GO item (y-axis). The size of each sphere indicated the number of genes overlapped with each GO item, and the color of each sphere indicated the significance level of enrichment, as shown in the color bar.


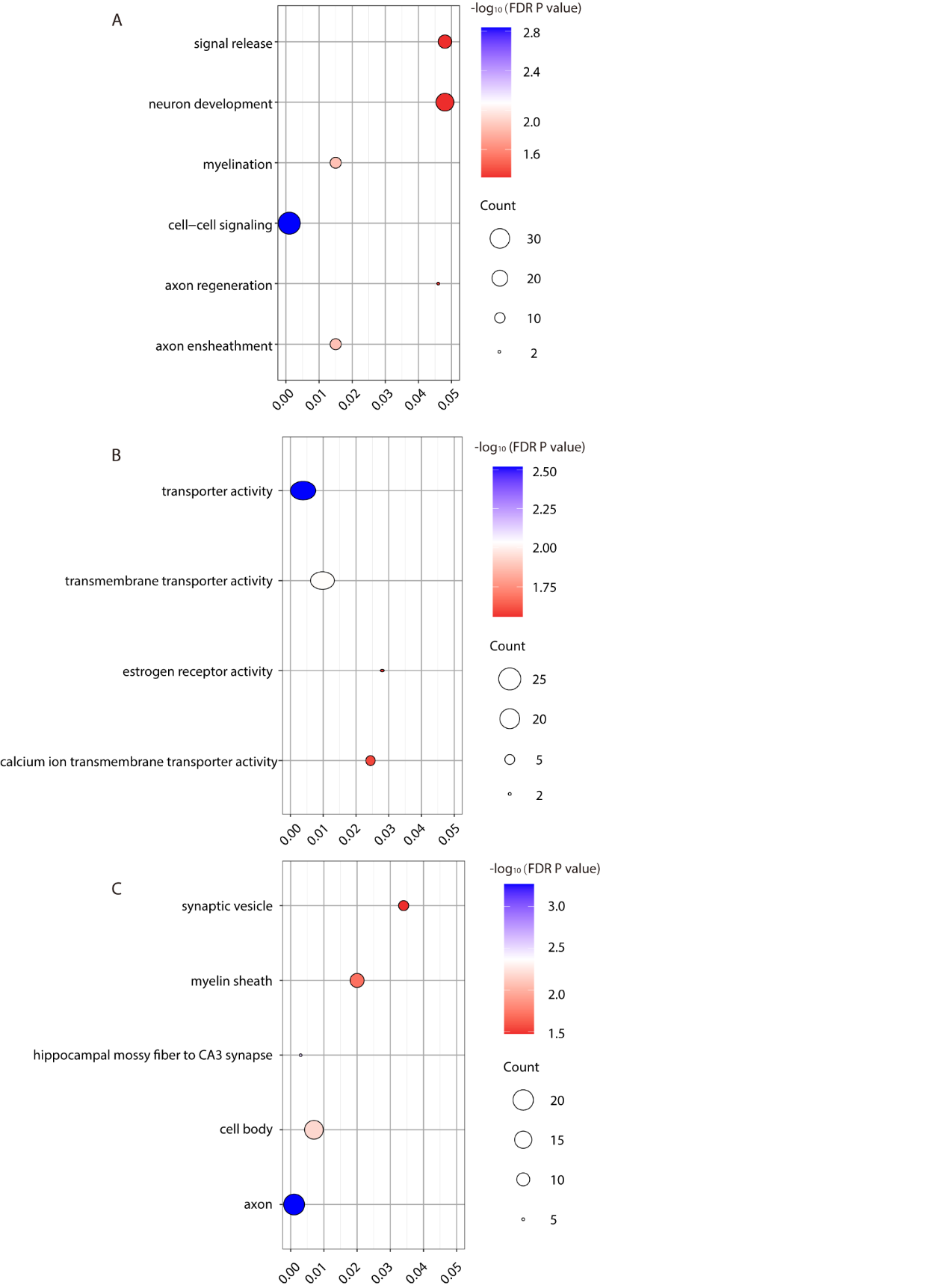


**Supplementary Figure 6.** **Gene enrichment of genes significantly associated with ReHo alterations in schizophrenia in** **Scale 3.** A. Significant gene ontology (GO) items of molecular function; B. Significant GO items of biological processes; C. Significant GO items of cellular components. The x-axis represented the P value of enrichment for each GO item (y-axis). The size of each sphere indicated the number of genes overlapped with each GO item, and the color of each sphere indicated the significance level of enrichment, as shown in the color bar.
